# Predictive value of glial fibrillary acidic protein for prognosis in patients with moderate and severe traumatic brain injury: a systematic review and meta-analysis

**DOI:** 10.1186/cc10905

**Published:** 2012-03-20

**Authors:** E Laroche, AF Turgeon, A Boutin, E Mercier, F Lauzier, R Zarychanski, L Moore, J Granton, P Archambault, F Lamontagne, F Rousseau, F Légaré, E Randell, J Lapointe, J Lacroix, D Fergusson

**Affiliations:** 1 Université Laval, Québec, Canada; 2 University of Manitoba, Canada; 3 University of Toronto, Ontario, Canada; 4 Université de Sherbrooke, Québec, Canada; 5 Memorial University, NewFoundland, Canada; 6 Université de Montréal, Québec, Canada; 7 Ottawa Hospital Research Institute, Ontario, Canada

## Introduction

Biomarkers have been proposed as potential prognostic indicators following a traumatic brain injury (TBI). Among those, glial fibrillary acidic protein (GFAP) has been one of the most studied. The objective of this study was to assess the prognostic value of GFAP levels in patients with moderate to severe TBI.

## Methods

We systematically searched Medline, Embase, Cochrane Central, Scopus, BIOSIS, TRIP, conference abstracts, bibliography of selected studies and narrative reviews. Cohort studies including ≥4 patients with moderate or severe TBI and reporting GFAP levels (sampled within the first 24 hours of care) from any biological tissue or fluid, and mortality or Glasgow Outcome Scale (GOS), were eligible. Two independent reviewers screened all citations, selected eligible studies and extracted data using a standardized data extraction form. Pooled results from random effect models are presented using geometric mean ratios (GMRs). I^2 ^tests were used to measure statistical heterogeneity.

## Results

We retrieved 4,709 citations and eight studies were deemed potentially eligible. Among those, one was found to be a duplicate publication. Seven studies were thus included (*n *= 404). Four studies presented data on mortality (3 or 6 months) and four studies used the GOS (6 or 12 months) as an outcome measure. We found significant associations between serum GFAP levels and mortality in pooled analysis of three studies (GMR 14.73 (95% CI 5.93 to 34.12); I^2 ^= 79%), and between GFAP and GOS ≤3 in three studies (GMR 8.80 (95% CI 3.94 to 19.66); I^2 ^= 77%). Two studies could not be used in pooled analyses: one presented means of GFAP levels from multiple samplings over time (GMR 1.98 (95% CI 1.06 to 3.70)) while the other presented the highest peak levels of GFAP during the acute phase of care (GMR 3.20 (95% CI 1.82 to 5.65)).

## Conclusion

Serum GFAP levels following TBI were significantly higher in patients showing an unfavourable prognosis (death or GOS ≤3). The small number of studies included precluded further exploration of statistical heterogeneity. More investigations of the association between serum GFAP levels and prognosis following TBI are needed before recommending for routine use for neuroprognostication.

